# Fluid-solid-heat coupling deformation analysis of the valve trims in a multistage pressure reducing valve

**DOI:** 10.1371/journal.pone.0263076

**Published:** 2022-01-25

**Authors:** Dongtao Xu, Changrong Ge, Ying Li, Yuejuan Liu

**Affiliations:** School of Mechanical Engineering & Automation, University of Science and Technology Liaoning, Liaoning Anshan, China; Tongji University, CHINA

## Abstract

A multistage pressure reducing valve with specially designed pressure reducing components is presented in this paper. As the deformation of the valve trims under fluid-solid-heat coupling has an important influence on the operation reliability of the valve, a numerical simulation is carried out to analyse the flow field characteristic in the valve and radial deformation of the valve trims using the ANSYS software. And a deformation experiment is designed to validate the deformations of the valve trims at high temperature of 693.15 K. The results indicate that the simulation results agree well with the experimental data. Moreover, it is found that the temperature field has the most significant influence on the deformation of the valve trims, the radial deformations of the matching surface vary from 0.439 to 0.442 mm. And the radial deformations caused by other factors vary from 0.005 to 0.015 mm. In addition, as a novel indicator, the clearance after deformation of the matching surface is used to evaluate the operation reliability of the valve. By using the GAP function in ANSYS static module, the clearances of the matching surface are obtained at different openings under the condition of fluid-solid-heat coupling, further indicating that the initial clearance between the valve plug and inner sleeve should be greater than 0.014 mm to ensure the operation reliability of the valve.

## Introduction

In modern industrial production, the regulating valve is an indispensable component in a control system, as it can regulate the flow rate and stabilize fluid pressure [1–3]. This valve is widely used in petrochemical, water conservancy, energy and other industrial sectors. With the development of science and technology, to improve the reliability of control systems, higher requirements are proposed for the design rationality of throttle components in regulating valves [4]. To ensure the operation reliability of the valves under the influence of high temperature and high pressure fluid, it is important to design an appropriate fit clearance between the trims. If the fit clearance is large, the overflow is serious and the flow rate is difficult to control. If the fit clearance is small, the deformations of the valve trims caused by high temperature and pressure fluid lead to the stuck phenomenon between the matching surface [5–7]. For the deformations of the valve trims, some scholars mostly use the finite element method to analyze the deformations due to the difficulty of internal measurement. S.V. Angadi studied the reliability of solenoid valves and established a multi-physical field model to carry out the finite element simulation analysis on temperature distribution, stress, thermal deformation and thermal stress [8, 9]. Marek A. studied the material behavior of a steam valve under mechanical and thermal loads, and found that the change of local stress caused by non-uniform temperature field was an important factor leading to metal fatigue [10]. Yi H K et al. analyzed the stress and strain of double-layer pipes under the condition of temperature change, which provided theoretical basis for the reliability evaluation in the double-layer pipes [11]. Yan J carried out numerical calculation on the influence of temperature rise on material properties of hydraulic spool valves. The results showed that thermal deformation of the spool was the main factor leading to failure of the hydraulic spool valves [12]. Liu X H analyzed the relationship between radial clearance of the valve trims and media temperature in a slide valve, aimed for preventing the deadlock of the valve trims caused by temperature increasing in the hydraulic spool valves [13]. Lv X T discovered the relationship between medium temperature, solid temperature and thermal deformation of hydraulic slide valves by simulation, which effectively prevented the stuck phenomena in the valves caused by the radial deformation in the spool [14].

In this paper, a multistage pressure reducing valve is presented. The novel pressure reducing components can be designed to not only satisfy the flow characteristics but also have good effects on reducing pressure. Fluid-solid-heat coupling field is simulated using the ANSYS workbench module. The deformations of the valve plug and inner sleeve are simulated and the influence of each physical field on the deformations of the valve trims is analyzed. An experiment is designed, which only considers the influence of the temperature field on the deformations of the valve trims. The experimental data is compared with the simulation data to verify the correctness of the simulation. Finally, the clearances after deformation of the matching surface are obtained under the condition of fluid-solid-heat coupling using the GAP function in the ANSYS static module. Setting an appropriate initial clearance of the matching surface is always a difficult problem in design. To ensure the operation reliability of the valve, the initial clearance of the valve trims is usually designed to be large. However, the fluid flows through the clearance, the overflow seriously affects the flow rate of the valve. The clearance after deformation can be directly used as the index to design the initial clearance of the matching surface. A suitable initial clearance can be found, so that the valve has good operation reliability and the overflow can be effectively reduced.

## Structural design of the multistage pressure reducing valve

### Structural description of the multistage pressure reducing valve

**Fig 1:**
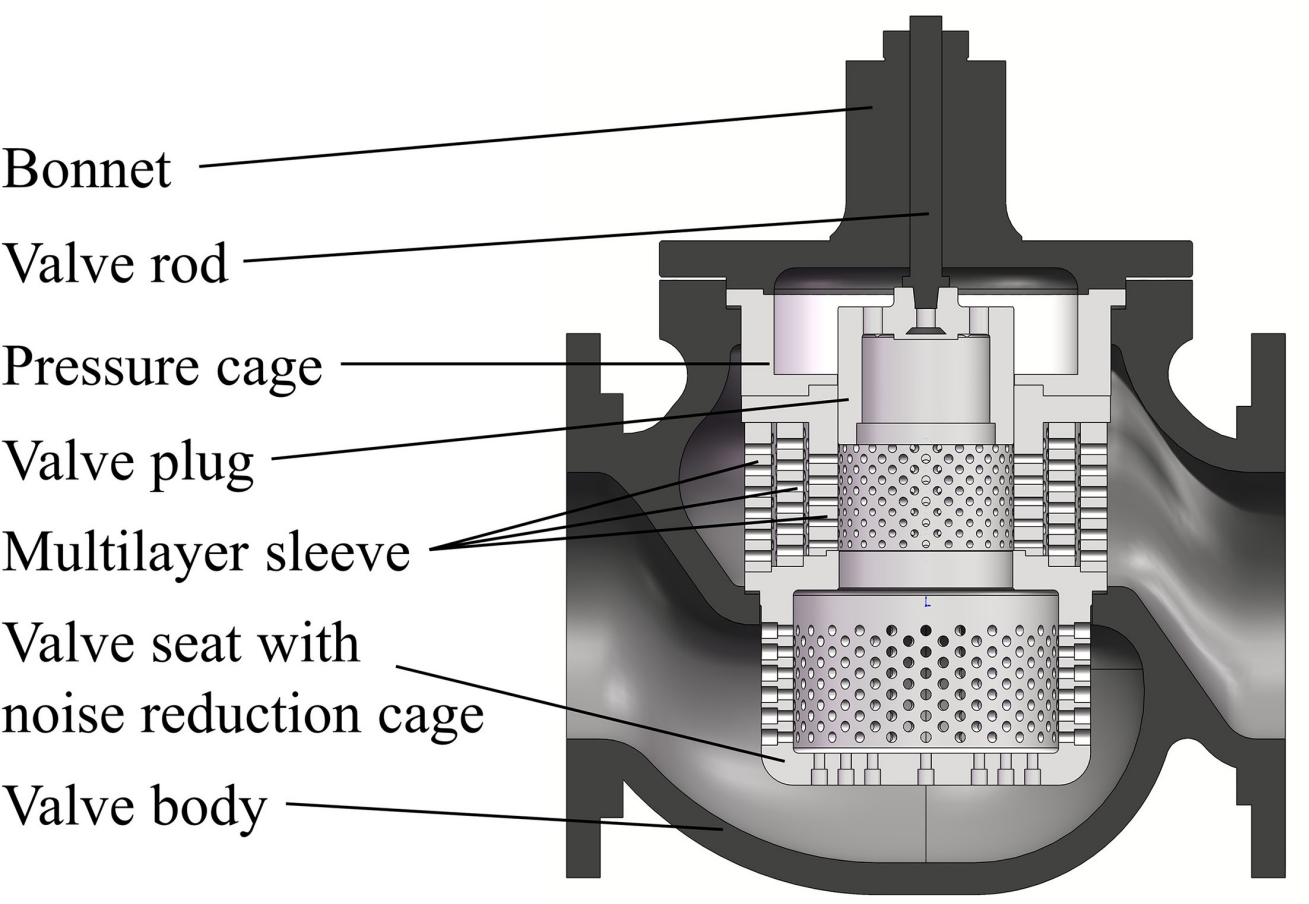
Multistage pressure reducing valve.

The structure of the multistage pressure reducing valve is shown in Fig 1.

The valve consists of a valve body, valve seat with noise reduction cage, multilayer sleeve, valve plug, pressure cage, valve rod and bonnet. The valve rod driven by the external actuator is able to drive the valve plug to move up and down. In this manner, the orifices in the inner sleeve are exposed to form effective flow areas.

When the fluid flows through the pressure reducing components, the flow velocity of the fluid increases as the flow area decreases quickly, and the static pressure of the fluid drops. If the pressure difference of each stage pressure drop is greater than the blocked flow critical pressure difference, blocked flow will occur. The orifices of the multilayer sleeve can gradually reduce the pressure of the fluid in the valve, the large pressure difference can be broken down into several small pressure differences to prevent block flow [15, 16].

### Design of the pressure reducing components

The orifices in the multilayer sleeve can reduce pressure and control flow at each opening. The flow rate through the orifices is calculated according to hydromechanics. Based on the thickness of the multilayer sleeve, the orifices are typically thin-walled holes. According to the law of the conservation of energy, the total energy of the fluid at the inlet is equal to the total energy at the outlet. According to the Bernoulli equation, the energy of the fluid can be expressed as follows:

$$\frac{p_{1}}{\rho g}+\frac{\alpha_{1}v_{1}^{2}}{2g}+h_{1}=h_{2}+\frac{p_{2}}{\rho g}+\frac{\alpha_{2}v_{2}^{2}}{2g}+\xi \frac{v_{e}^{2}}{2g}$$ (1)

where the pressure difference of the fluid at the inlet and outlet: Δ*p* = *p*_1_-*p*_2_; *p*_1_ and *p*_2_ are the fluid pressure at the inlet and outlet, respectively; *h*_1_ and *h*_2_ are the potential energy of the fluid in the inlet and outlet, respectively; *v*_1_ and *v*_2_ are the flow velocity of the fluid flowing through the inlet and outlet, respectively; *ρ* is the density of the fluid; *α*_1_ and *α*_2_ are the kinetic energy correction coefficient at the inlet and outlet, respectively; (*v*_*e*_^2^/2*g*) is energy loss when the fluid flows through the orifice components; *ξ* is the flow resistance coefficient; *g* is the acceleration of gravity.

The nominal diameter of the valve is *d*. According to the continuity equation, the flow rate of the fluid flowing through the valve is constant, i.e., *A*_1_·*v*_1_ = *A*_2_·*v*_2_ (*A*_1_, *A*_2_ are the cross-sectional area at the inlet and outlet, respectively, and *A*_1_ = *A*_2_ = π*d*), so: *v*_1_ = *v*_2_. Thus, *α*_1_ = *α*_2_ = 1, and the fluid potential energy in the inlet and outlet is equal, i.e., *h*_1_ = *h*_2_. According to Eq (1), the flow velocity of the fluid flowing through the orifices is obtained as follows:

$$v_{e}=\frac{1}{\sqrt{\xi }}\sqrt{\frac{2\Delta p}{\rho }}$$ (2)

According to Eq (2), the sectional area of the orifices is *A*_*e*_, the flow rate of the fluid flowing through the orifices is obtained as follows:

$$Q=A_{e}v_{e}=\frac{A_{e}}{\sqrt{\xi }}\sqrt{\frac{2\Delta p}{\rho }}$$ (3)

Eq (3) shows the flow rate of the fluid is closely related to density *ρ* of the fluid, the flow area of the orifices *A*_*e*_, the pressure difference Δ*p*, and the flow resistance coefficient *ξ*. Therefore, according to Eq (3), the flow area can be obtained according to the flow rate at different openings. Therefore, the configuration of orifices on the sleeve can be designed.

The pressure reducing components are designed as a multilayer sleeve. The number of decompression stages *n* can be expressed as:

$$n=-3.851\mathrm{lg}(p_{2}/p_{1})$$ (4)

When the fluid flows through each stage pressure reduction component, the pressure difference ratio is:

$$p_{m}^{\prime }/p_{m-1}^{\prime }={\left(p_{2}/p_{1}\right)}^{\frac{1}{2}}$$ (5)

where $p_{m}^{\prime }$, $p_{m-1}^{\prime }$ are the inlet pressure and outlet pressure of the *m*th stage pressure reducing component, respectively.

The flow area of each stage pressure reduction component should satisfy the flow rate at a 100% opening, and combined with Eq (5), the flow area of each stage pressure reduction component can be obtained:

$$A=\frac{0.057\pi G}{C_{0}P_{m-1}\sqrt{\left(\frac{M}{ZT})(\frac{k}{k-1})[{\left(\frac{p_{m}^{\prime }}{p_{m-1}^{\prime }}\right)}^{\frac{2}{k}}-{\left(\frac{p_{m}^{\prime }}{p_{m-1}^{\prime }}\right)}^{\frac{k+1}{k}}\right]}}$$ (6)

where *G* is the mass flow rate of the fluid; *C*_0_ is the flow coefficient of the single-hole; *M* is the molar mass of the fluid; *Z* is the compression coefficient of the fluid; *T* is the fluid temperature; *k* is Adiabatic index.

The orifice configuration of the pressure reducing components can be designed by Eq (6).

The flow characteristic of the valve refers to the ratio between the relative flow rate and the relative stroke in the valve. The linear flow characteristic satisfies the proportional relationship between the relative flow rate and the relative stroke. The functional relationship can be described as:

$$q=\frac{Q}{Q_{\max}}=\frac{R-1}{R}\cdot l+\frac{1}{R}$$ (7)

where *q* is the relative flow rate; *Q* is the flow rate at a certain opening; *Q*_max_ is the flow rate at a 100% opening; *l* is the relative stroke; *R* is the adjustable ratio (1/50).

**Fig 2:**
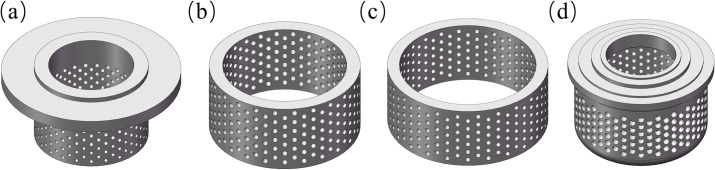
The pressure reducing components. (a) Inner sleeve. (b) Middle sleeve. (c) Outer sleeve. (d) Valve seat with noise reduction cage.

When the nominal diameter of the valve body is 250 mm, the inlet and outlet pressures are 4.1 and 0.5 MPa, respectively, and the valve is designed according to the linear flow characteristics (*C*_*v*_ = 360), the pressure reducing components are designed, as shown in Fig 2:

## Flow field simulation of the multistage pressure reducing valve

### Establishing the simulation model

Prior to simulation calculation, a virtual simulation 3D model of the multistage pressure reducing valve is established, as shown in Fig 1. The nominal diameter of the valve is Φ250 mm and the valve plug diameter is Φ165mm. According to the actual working conditions, the fluid in the valve is a superheated steam with temperature of 693.15 K. The Fluent module under the ANSYS workbench platform is used to perform the flow field simulation. The pressure at the inlet of the valve is 4.1 MPa, and the pressure at the outlet is 0.5MPa. The material of the valve trims is 15CrMo.

The RNG *k*-*ε* turbulence model is used for the flow field simulation in this paper. The RNG *k-ε* model is based on Standard *k-ε* model and is used to simulate the complex high curvature flow field [17–19]. In the complex flow field of the Newtonian liquid, the numerical simulation is more accurate when the RNG *k-ε* model is used but difficult to converge [20]. Variable parameters in the model are correlated with the fluid model, fluid flow state, and corresponding space coordinate system functions. The standard wall function is used, the couple algorithm and second-order windward format are chosen to accelerate convergence [21]. The above parameters can effectively simulate the flow field of the Newtonian fluid in the valve [22].

To simulate the flow field of the valve, the fluid model is divided into tetrahedral and hexahedron hybrid meshes using the ANSYS meshing tool. The grid independence is checked, and the reference value of the grid independence is based on the flow rate and the average flow velocity at the outlet of the valve at a 100% opening. The test data is shown in Table 1:

**Table 1:** Fluid grid independence test data.

Number of grid cells	Outlet velocity (m/s)	Outlet flow (m^3^/h)
3951741	15.698	2772.558
4263695	15.709	2773.955
5177818	15.805	2789.258
6361212	15.811	2788.952

According to Table 1, when the number of grids increases from 5177818 to 6361212, the outlet velocity increases by 0.038% and the outlet flow rate decreases by 0.01%. When the number of grids is more than 5177818, the change in velocity and flow rate at the outlet can be ignored. Further refinement of the finite element mesh has no significant effect on the simulation results.

### Numerical simulation of the flow field

**Fig 3:**
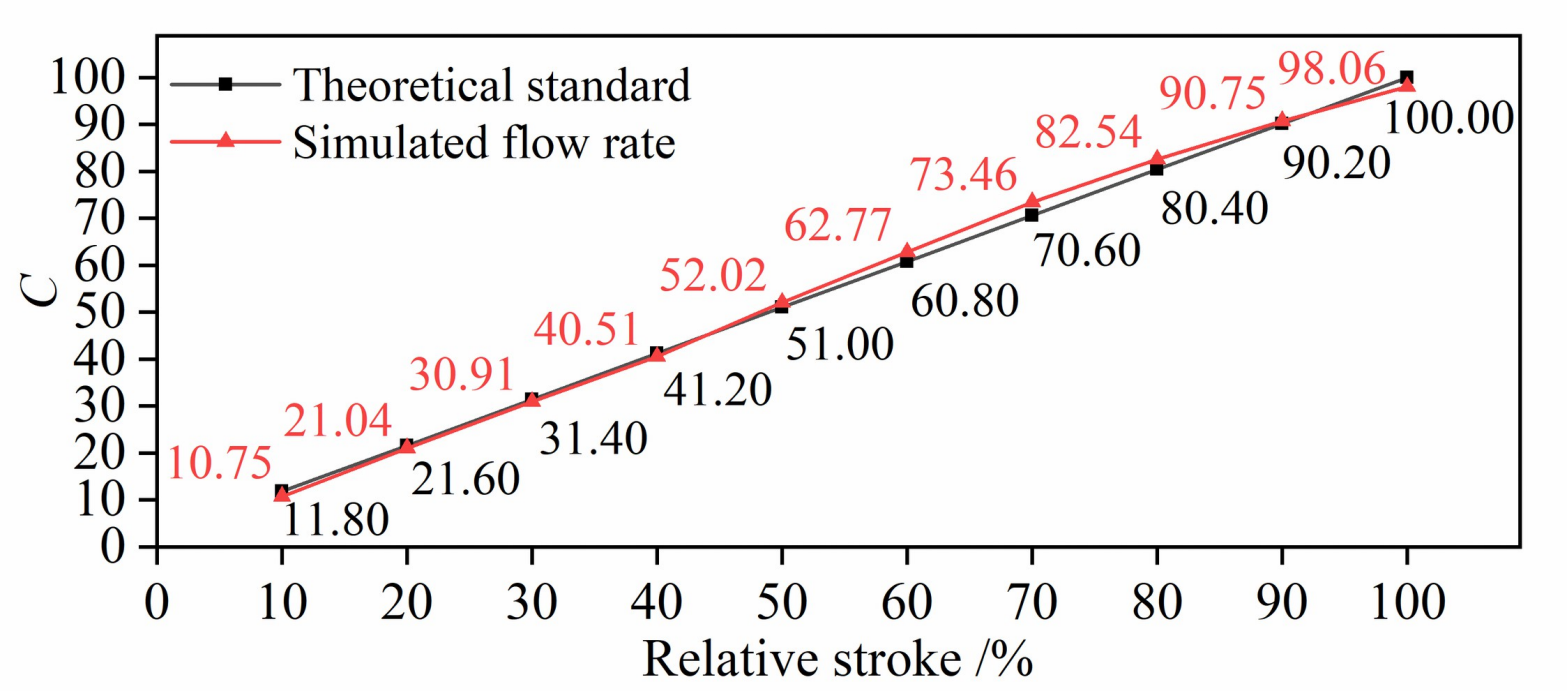
Comparison of simulated flow rate with standard data.

The maximum stroke of the valve plug is 100 mm. The flow rate at the outlet of the valve is monitored. After the iterative calculation, the circulation volume of the valve is obtained. The flow rate obtained by simulation is compared with the theoretical flow rate, as shown in Fig 3.

*C* is the relative flow coefficient and is dimensionless; it is the ratio of the flow rate at a certain opening to the circulation volume (*Cv*). Fig 3 shows that the designed valve meets the linear flow characteristics (*Cv =* 356.32) and the flow rate obtained by the simulation at each opening is within the allowable error range. The maximum error is at a 70% opening, and the error value is only 4.06%.

**Fig 4:**
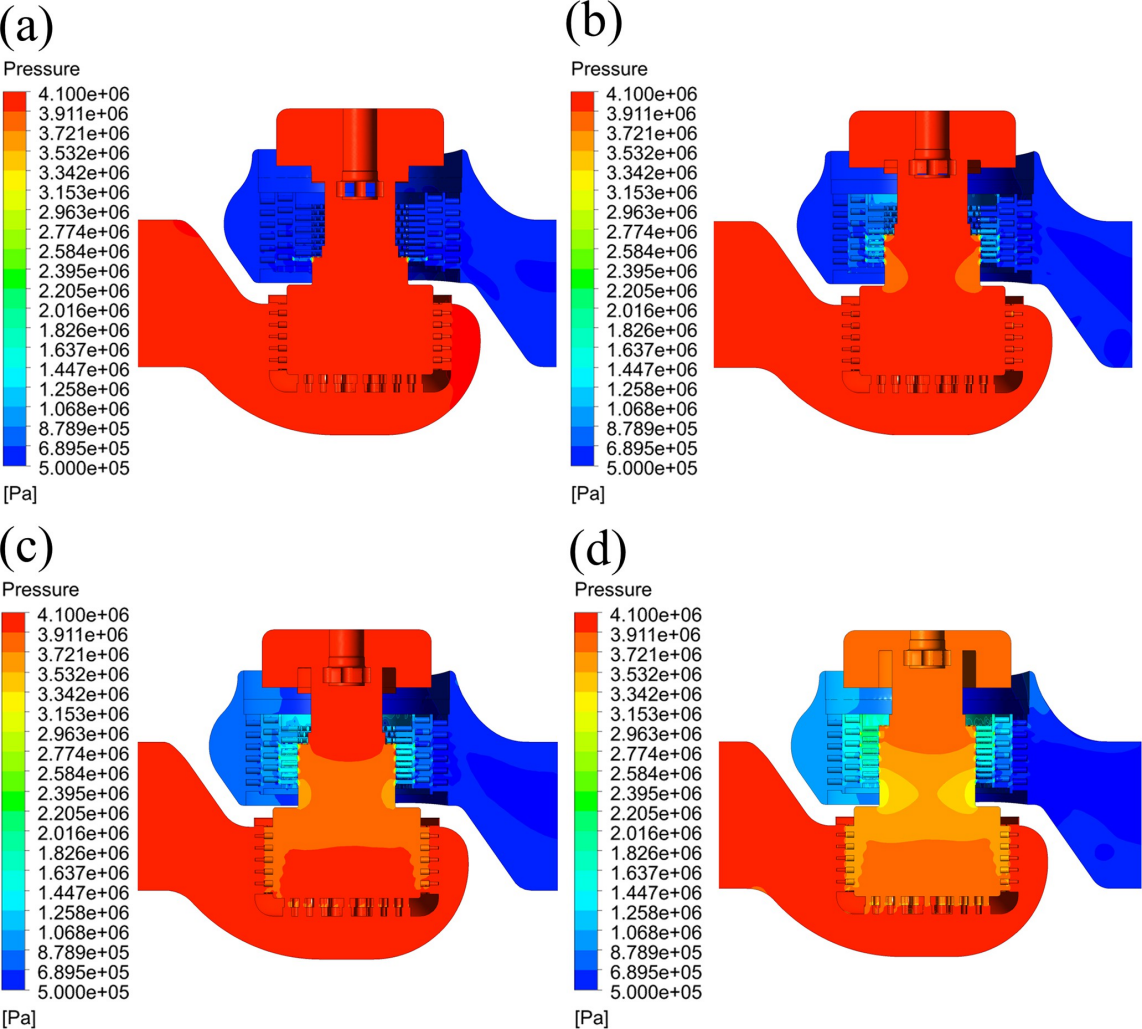
Pressure distribution of the flow field at different opening. (a) 10% opening. (b) 50% opening. (c) 70% opening. (d) 100% opening.

After the simulation, in the CFD-Post unit, the pressure distribution of the flow field at different openings is obtained, as shown in Fig 4.

At a 10% opening, only some orifices in the lower part of the inner sleeve are in the flow state. The fluid pressure drops significantly when the fluid flows through the orifices. At this moment, the flow rate is relatively small, and the fluid pressure does not change significantly as the fluid flows through the valve seat with noise reduction cage and the outer sleeve. At openings of 50%, 70%, and 100%, when the fluid flows through the multilayer sleeve, the static pressure of the fluid drops significantly. The pressure distributions of the flow field conform to the designed pressure drop rule. The orifices of the inner sleeve not only control the flow rate well but also have a pressure-reducing function.

## Deformation analysis of the valve trims

In the flow field, the valve trims can deform due to the mutual extrusion, the temperature field, and the fluid field in the valve. In the axial direction of the valve rod, due to the addition of flexible sealing materials, the deformations of the valve trims have little impact on the operation reliability of the valve. In the radial direction of the valve rod, when the radial deformations of the valve plug are larger than those of the inner sleeve at a certain position, the stuck phenomenon may occur.

### Radial deformation analysis of the valve trims in the fluid-solid-heat coupling field

**Fig 5:**
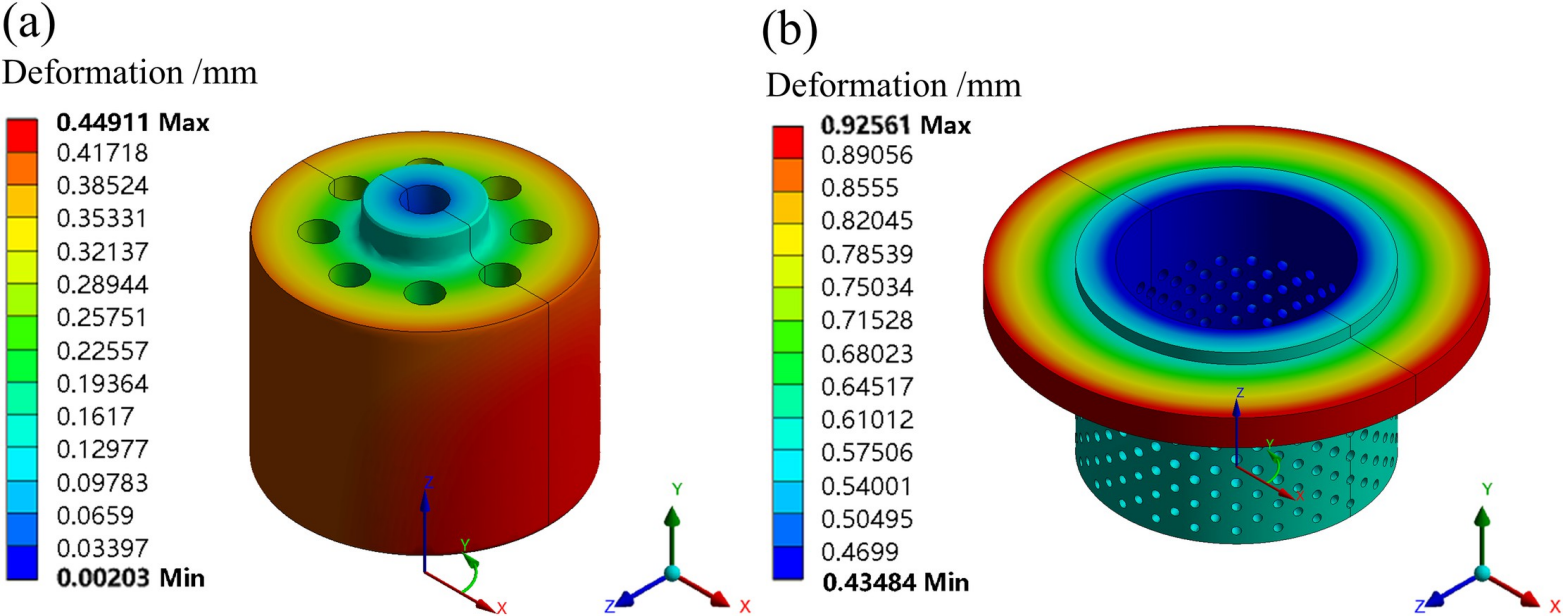
Radial deformation of the inner sleeve and valve plug at a 50% opening. (a) Valve plug. (b) Inner sleeve.

In the static module, the material properties are set up and the displacement constraints are added at both ends of the piping system and at the top of the valve rod. In the post-processing module, the cylindrical coordinate system is applied, and the *Y*-axis is parallel to the valve rod axis, the *X*-axis increment is the radial deformation. The contact relations and initial clearances of the matching surfaces are set. The outer surface of the valve plug and the inner surface of the inner sleeve are a pair of matching surfaces. The assembly dimensions are designed in accordance with $\Phi 165D9_{-0.145}^{+0.245}/h9_{-0.040}^{0}$. The initial clearance is set to 0.073 mm. The radial deformation distributions of the inner sleeve and valve plug are simulated by ANSYS workbench under the condition of fluid-solid-heat coupling, as shown in Fig 5.

Fig 5 shows the radial deformation of the valve plug and inner sleeve at a 50% opening. The deformation is positive, it means that the radial size increases. According to Fig 5, the radial dimensions of the valve trims tend to increase, the maximum deformation of the valve plug is distributed on the outer cylindrical surface, its value is 0.449 mm. The maximum deformation of the inner sleeve is distributed on the upper cover, its value is 0.926 mm, the minimum deformation is distributed on the inner cylinder, its value is 0.435 mm. The maximum deformation on the outer surface of the valve plug is greater than the minimum deformation on the inner surface of the inner sleeve.

**Fig 6:**
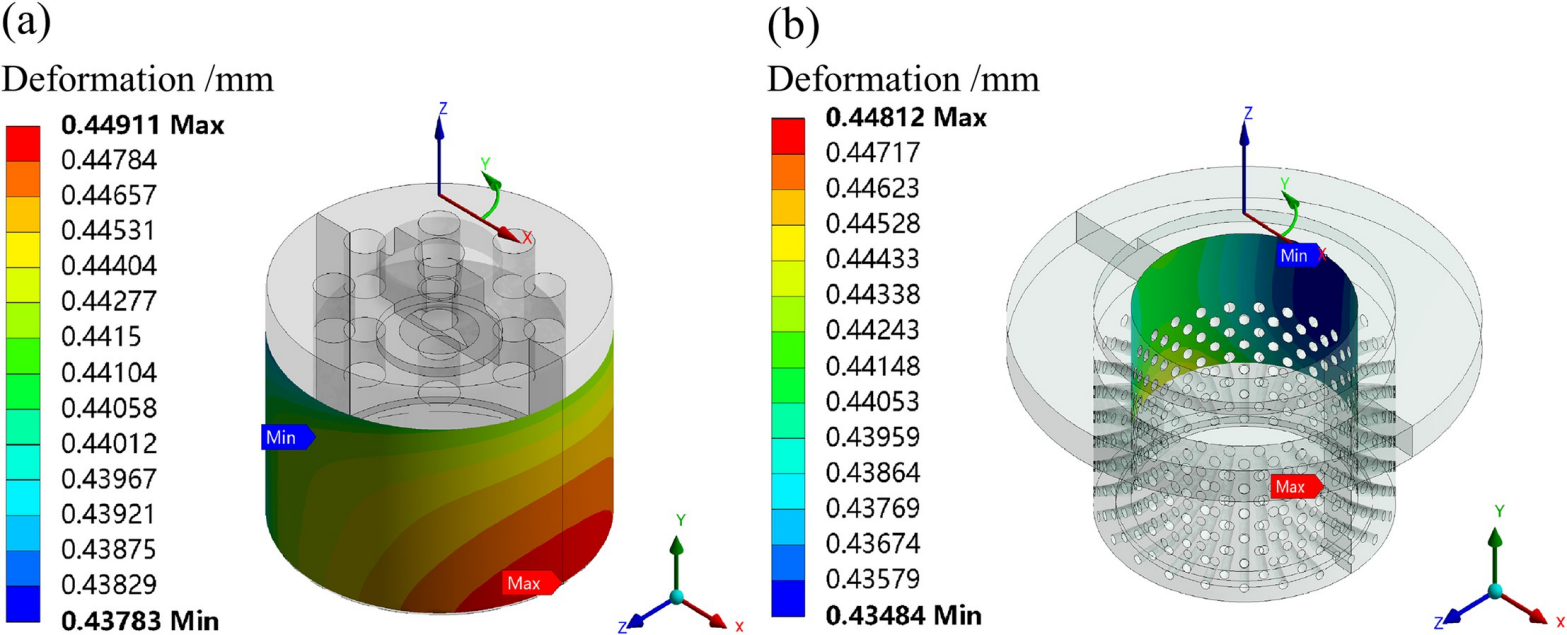
Radial deformation of the matching surfaces at a 50% opening. (a) Valve plug. (b) Inner sleeve.

The radial deformation distributions of the matching surfaces are simulated at a 50% opening under the condition of fluid-solid-heat coupling, as shown in Fig 6.

The maximum deformation of the valve plug matching surface is distributed on the lower part of the outer surface, its value is 0.449 mm. The minimum deformation is on the upper part, its value is 0.438 mm. The maximum deformation of the inner sleeve matching surface is at the upper part of the inner surface, its deformation value is 0.448 mm. The maximum deformation of the valve plug matching surface is greater than the minimum deformation of the inner sleeve matching surface.

### Influence degree of each physical field on radial deformation of valve internals

**Fig 7:**
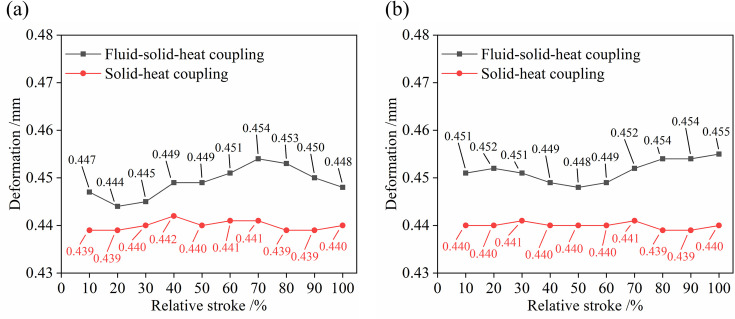
Comparison curves of the deformations of the matching surface. (a) Valve plug. (b) Inner sleeve.

The influence of different physical fields on the deformations of the valve trims is analyzed when the initial clearance is set to 0.073 mm. The maximum and minimum deformation of the matching surface are simulated under fluid-solid-heat and solid-heat coupling at different openings, as shown Fig 7.

When the fluid temperature is 693.15K, the temperature field has the most significant influence on the deformations of the valve trims, and the deformations vary from 0.439 to 0.442 mm. The vertical coordinates differences of the two curves are the influence of the flow field pressure on the radial deformation of the matching surface, and the deformation values vary from 0.005 to 0.015 mm. The flow field in the valve also has some small influence on the valve trims.

### Deformation experiment of the valve trims under the temperature field

**Fig 8:**
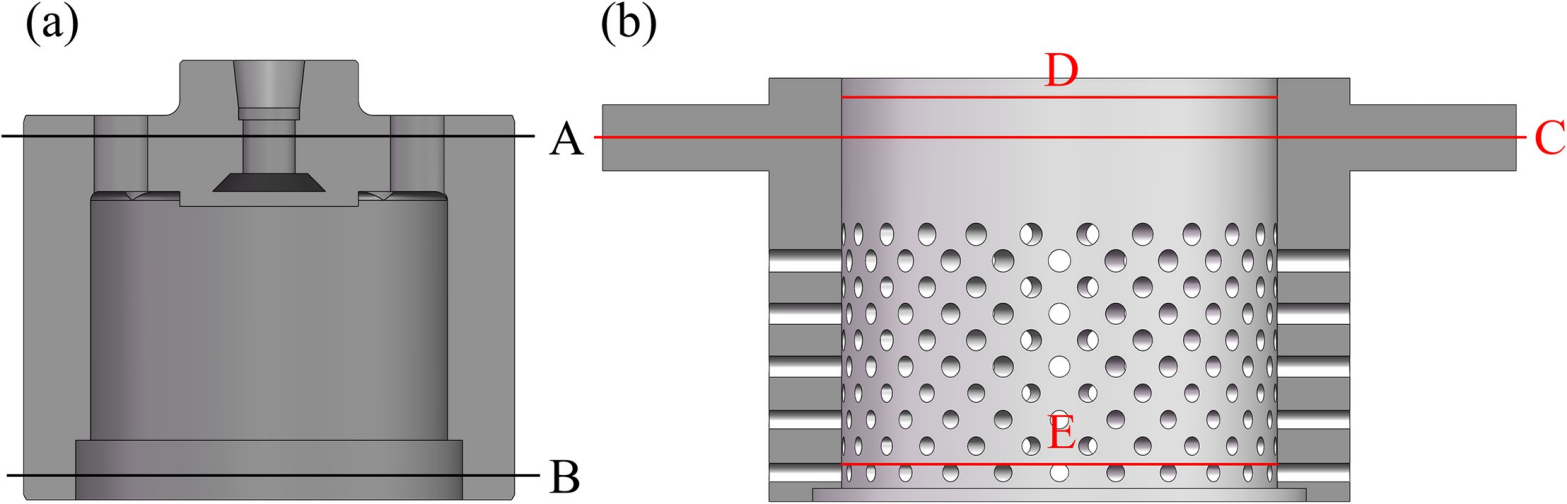
Measuring location of the valve trims. (a) Valve plug. (b) Inner sleeve.

To verify the correctness of the simulation results, a high-temperature deformation experiment on the valve plug and inner sleeve is conducted. The influence of the temperature field on the valve plug and inner sleeve is considered in the experiment. Their material of the valve plug and inner sleeve is 15CrMo. The structure and dimensions are the same as those of the simulation models. The measuring locations are located at the external diameter at the location A and B of the valve plug and at the location C of the inner sleeve, and internal diameter of location D and E of the inner sleeve, as shown in Fig 8.

The dimensions at different locations on the circumference of the valve plug and inner sleeve are measured three times at room temperature using a vernier calipers, and then averaged. The valve plug and inner sleeve are placed in an industrial heating furnace and slowly heated to 693.15 K and kept the temperature for 5 minutes to ensure uniform heating. Then, the valve plug and inner sleeve are measured with the protection of the protective gear and the results are shown in Table 2.

**Table 2:** Measurement data of different locations of valve trims.

	Unit: mm	Location A	Location B	Location C	Location D	Location E
Temperature 298.15 K	Measured value	164.98	164.99	345.86	165.14	165.13
		164.99	164.98	345.92	165.13	165.13
		164.96	164.98	345.88	165.11	165.14
	Average value	164.98	164.98	345.88	165.13	165.13
Temperature 693.15 K	Measured value	165.89	165.85	347.73	166.01	166.00
		165.88	165.86	347.72	166.01	166.01
		165.88	165.87	347.71	166.02	166.01
	Average value	165.88	165.86	347.72	166.01	166.01
Deformation value		0.45	0.44	0.92	0.44	0.44

Table 2 shows that the measured values of the valve trims at different locations under the room temperature (298.15 K) and high temperature (693.15 K). The radial dimensions of the valve plug and inner sleeve increase uniformly under the influence of the temperature field. The deformation at location C of the inner sleeve is 0.92 mm. The radial deformations of the matching surface are about 0.44 mm. The deformation values obtained by the experiment are close to those obtained by the simulation analysis. It shows that the simulation data is reliable.

### Analysis of the radial clearance after deformation under the condition of fluid-solid-heat coupling

**Fig 9:**
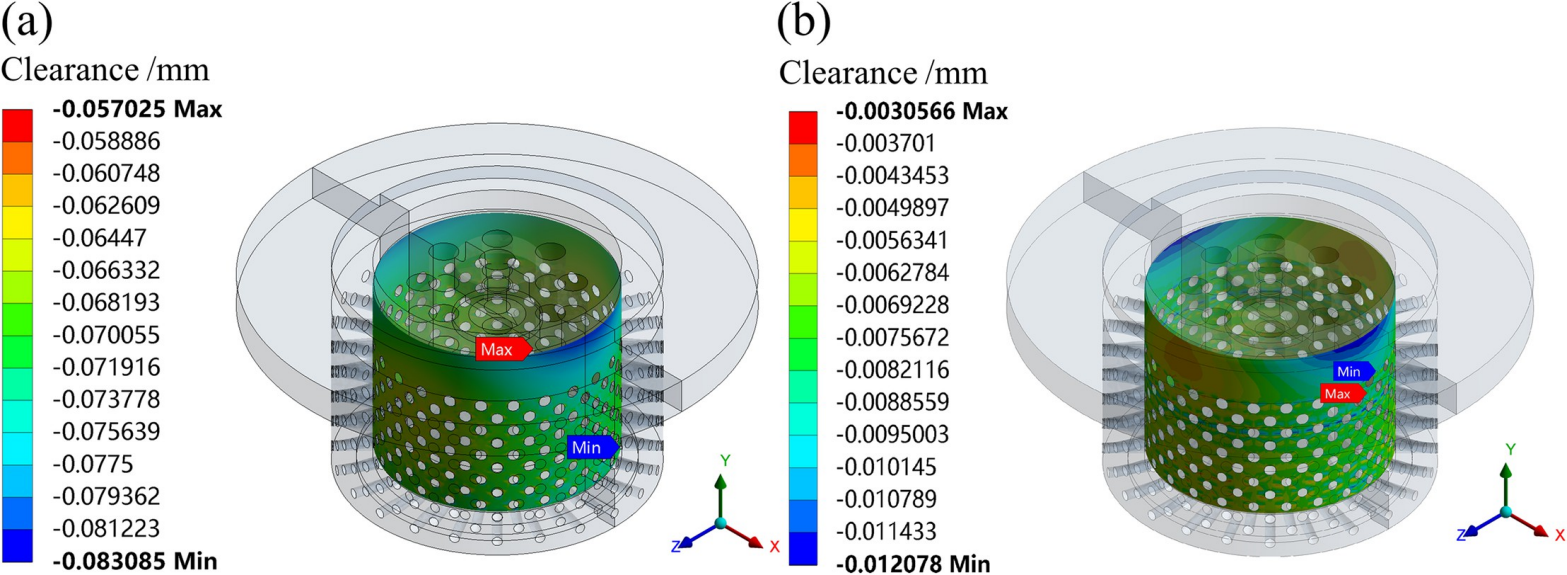
Clearances after deformation of the matching surface at a 10% opening. (a) Initial clearance: 0.073 mm. (b) Initial clearance: 0.010 mm.

**Fig 10:**
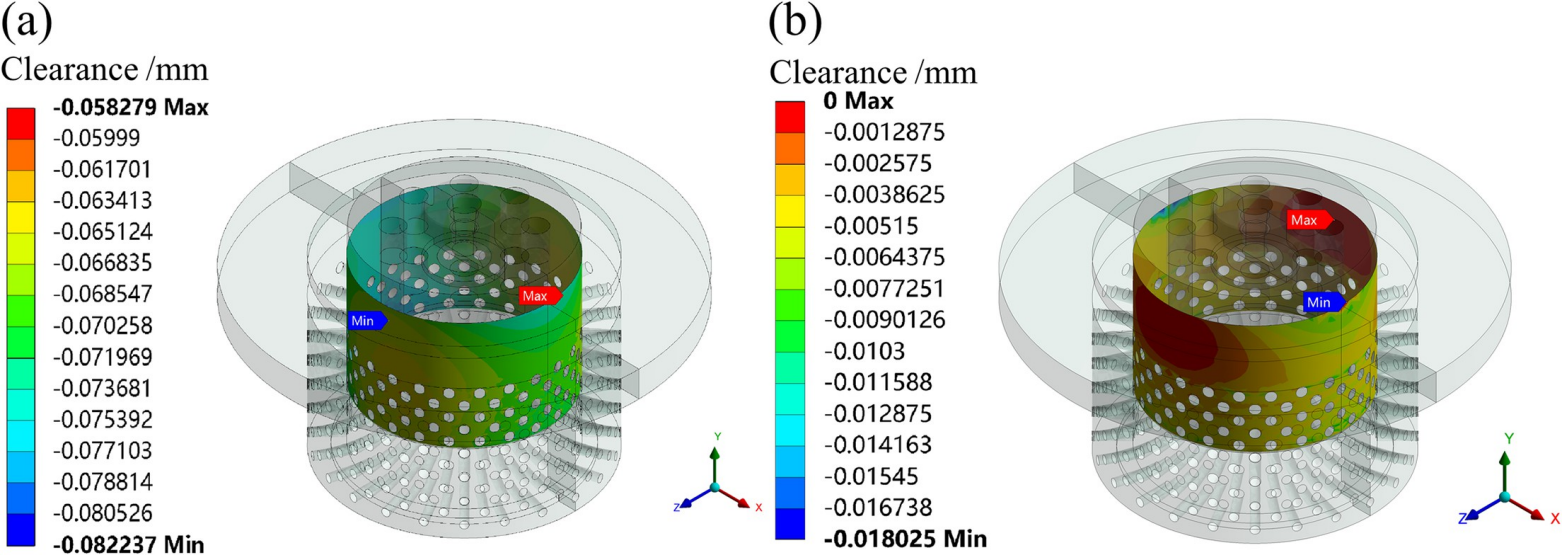
Clearances after deformation of the matching surface at a 50% opening. (a) Initial clearance: 0.073 mm. (b) Initial clearance: 0.010 mm.

**Fig 11:**
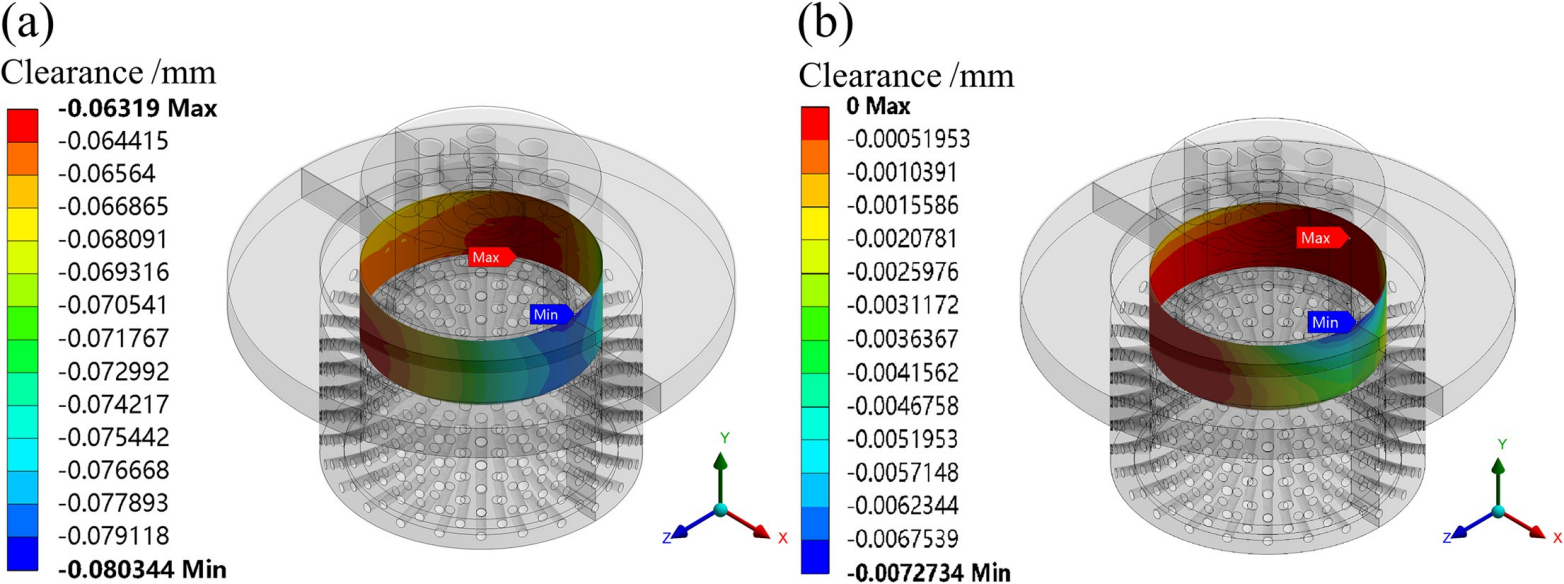
Clearances after deformation of the matching surface at a 100% opening. (a) Initial clearance: 0.073 mm. (b) Initial clearance: 0.010 mm.

The simulation results show that the maximum deformation of the valve plug matching surface is greater than the minimum deformation of the inner sleeve matching surface under the condition of fluid-solid-heat coupling. So, it is very important to set an appropriate initial clearance in design. If the initial clearance is small, the stuck phenomenon may occur. If the initial clearance is large, the overflow is serious. To analyze the operation reliability of the valve under the condition of fluid-solid-heat coupling, the radial clearances after deformation of the matching surface are simulated at different openings using the GAP function in the contact tools of the ANSYS statics module. When the initial clearances are set to 0.073 and 0.010 mm, respectively, the radial clearances after deformation of the matching surface are simulated, as shown in Figs 9–11.

If the simulation value is negative, it indicates that there is radial clearance between the inner sleeve and valve plug. If the simulation value is 0, it indicates that the inner sleeve is in contact with the valve plug, the extrusion has occurred on matching surface. At a 10% opening, when the initial clearance is set to 0.073 mm, the simulation shows that the minimum clearance after deformation of the matching surface is 0.057 mm; the initial clearance is set to 0.010 mm, the minimum clearance is 0.031 mm. The stuck phenomenon does not occur.

At a 50% opening, the initial clearances are set to 0.073 and 0.010 mm, respectively. The simulation shows that the minimum clearances of the matching surface are 0.058 and 0 mm, respectively. Therefore, when the initial clearance is set to 0.010 mm, the stuck phenomenon has occurred.

At a 100% opening, the initial clearances are set to 0.073 and 0.010 mm, respectively. The simulation shows that the minimum clearances of the matching surface are 0.056 and 0 mm, respectively. It can be seen from Fig 11 that the stuck phenomenon has occurred on the matching surface when the initial clearance is set to 0.010 mm.

Obviously, when designing the initial clearance between the valve plug and inner sleeve, the initial clearance value should be greater than 0.010 mm. However, if the clearance is too large, the overflow is serious, and the flow characteristics of the valve cannot be guaranteed.

The initial clearances are set to 0.011, 0.012, 0.013 and 0.014 mm, respectively, and the corresponding simulation analyses are carried out. The simulation results are shown in Table 3.

**Table 3:** The simulation clearances at different openings when different initial clearances are set.

Initial clearance /mm	At 10% opening simulation clearance /mm	At 50% opening simulation clearance /mm	At 100% opening simulation clearance /mm
0.011	-0.005	0	0
0.012	-0.006	0	0
0.013	-0.006	-0.001	0
0.014	-0.007	-0.001	-0.001

When the valve is at a large opening, it is easier to get stuck in spite of the smaller matching area. When the initial clearance value is set to 0.014 mm, the simulation clearances are negative at openings of 10%, 50% and 100%, so the stuck phenomenon does not occur; when the initial clearance is set to 0.013 mm, the simulation clearance at a 100% opening is 0; when the initial clearances are set to 0.011 and 0.012 mm, respectively, the simulation clearances at openings of 50% and 100% are 0. Therefore, it can be concluded that the minimum initial clearance between the valve plug and inner sleeve should be greater than 0.014 mm to ensure the operation reliability of the valve.

## Conclusions

In this study, a multistage pressure reducing valve with novel pressure reducing components was proposed. Based on the fluid-solid-heat coupling model validated by the high-temperature deformation experiment, the flow field, radial deformation and clearance after deformation of the multistage pressure reducing valve were analyzed. Results showed that the valve could not only satisfy the linear flow characteristics (*Cv* = 356.32) but also had a good pressure reducing function, and the temperature field had the most significant influence on the deformation of the valve trims. Furthermore, the radial deformation caused by temperature field accounted for about 97% of the total radial deformation under the condition of fluid-solid-heat coupling, and the other factors accounted for about 3% when the fluid temperature was 693.15 K. By analyzing the fluid-solid-heat coupling deformation of the valve trims, an optimal scheme of the initial clearance of the matching surface was presented. It is found that the valve not only had good operation reliability but also limited the overflow to the maximum extent when the initial clearance was set to 0.014 mm.

## Supporting information

S1 File Original data of tables. (ZIP)
